# Premature Dropout From Psychotherapy: Prevalence, Perceived Reasons and Consequences as Rated by Clinicians

**DOI:** 10.32872/cpe.6695

**Published:** 2022-06-30

**Authors:** Niclas Kullgard, Rolf Holmqvist, Gerhard Andersson

**Affiliations:** 1 Department of Behavioural Sciences and Learning, Linköping University, Linköping, Sweden; 2 Department of Biomedical and Clinical Sciences, Linköping University, Linköping, Sweden; 3 Department of Clinical Neuroscience, Karolinska Institute, Stockholm, Sweden; Philipps-University of Marburg, Marburg, Germany

**Keywords:** premature dropout, psychotherapy dropout, psychotherapy, therapeutic alliance

## Abstract

**Background:**

Why clients discontinue their psychotherapies has attracted more attention recently as it is a major problem for many healthcare services. Studies suggest that dropout rates may be affected by the mode of therapy, low-quality therapeutic alliance, low SES, and by conditions such personality disorders or substance abuse. The aims of the study were to investigate what happens in therapies which end in a dropout, and to estimate how common dropout is as reported by practicing clinicians.

**Method:**

An online questionnaire was developed and completed by 116 therapists working in clinical settings. They were recruited via social media (Facebook and different online psychotherapy groups) in Sweden and worked with Cognitive Behavioural Therapy (CBT), Psychodynamic Therapy (PDT), Interpersonal Psychotherapy (IPT) and Integrative Psychotherapy (IP).

**Results:**

Psychotherapists rated the frequency of premature dropout in psychotherapy to be on average 8.89% (MD = 5, SD = 8.34, Range = 0-50%). The most common reasons for a dropout, as stated by the therapists, were that clients were not satisfied with the type of intervention offered, or that clients did not benefit from the treatment as they had expected. The most common feeling following a dropout was self-doubt.

**Conclusion:**

In conclusion, premature dropout is common in clinical practice and has negative emotional consequences for therapists. Premature dropout may lead to feelings of self-doubt and powerlessness among therapists. The therapeutic alliance was mostly rated as good in dropout therapies. Further research is needed to validate the findings with data on the prevalence and subjective reasons behind a dropout from point of view of clients.

## Highlights

- The dropout rate was estimated to 8.89% by the therapists in this study.
- The psychotherapists believe that reasons as to dropout was that clients were not satisfied with interventions in therapy.
- The most common feeling following a dropout was self-doubt.
- The therapeutic alliance was generally rated as good in therapies resulting in dropout.

Dropout from psychotherapy has been defined as “termination of the treatment without fulfilment of the therapeutic goals, without attainment of the full therapeutic benefit that would have been possible with normal termination of the therapy or without completion of the full scope of the therapy” (Swift & Greenberg, 2012). There is a significant amount of variation on how to operationalize dropout, for example when it is meaningful to use dropout as a description of what happened in a therapy (Garfield, 1994; Hatchett & Park, 2003; Swift et al., 2009; Swift & Greenberg, 2012). One way to operationalize dropout is to consider anyone who do not attend a special number of sessions as a dropout. The idea is that clients need to attend a minimal number of sessions in order to improve (Lambert, 2007). Another operationalization is failure to complete a specific treatment protocol. In this definition anyone who fails to complete a full treatment protocol is considered a dropout. A third operationalization is based on missed sessions. This approach suggests that anyone who misses or fails to reschedule sessions is considered a dropout. Another fourth approach is to let the therapist decide if the client has prematurely dropped out or not. A final approach is to define a dropout when a client terminates prior to a reliable improvement has occurred and prior to obtaining an outcome score within the normal range (Hatchett & Park, 2003). There are both positive and negative aspects of all these operationalizations. While number of sessions, missed sessions and failure to follow a treatment protocol are relatively easy to assess they do not say anything about actual change or improvement. It is problematic to classify a client as a dropout when attending few sessions and showing major improvement when a client who attends all scheduled sessions but do not engage in the therapy and show no improvement will not be defined as a dropout.

When using therapists’ judgement there is a considerable risk that the judgment is biased or even flawed (Garb, 2005; Grove et al., 2000). Despite numerous studies there is *no consensus* regarding the definition of dropout. For example, as mentioned it is possible to drop out from a therapy while still reaching the treatment goals. The term premature suggests that the therapy is terminated *before* the goals of the treatment are obtained. While there are premature terminations of therapy that are agreed upon, not turning up and ending therapy *without explanation* or any notice can be a major problem. For example, clients may not get the treatment they need, and therapists and services are disrupted (for example when trying to locate the client). In a meta-analysis of 125 psychotherapy studies, Wierzbicki and Pekarik (1993) estimated that about 47% of the therapies resulted in a unilateral dropout. However, Swift and Greenberg (2012) reported dropout rates across methods and disorders at approximately 19.7%, and unilateral dropouts ranged between 0 to 74% (*M* = 19.7%) (Swift & Greenberg, 2012, 2014). Most studies included in these reviews were clinical trials on adult clients who were participants in studies in which both clients and methods had been carefully selected. Thus, there may be differences in reasons behind dropout in therapies conducted in clinical practice and in clinical trials depending on the definition of dropout or the context (for example interviews, questionnaires, videorecording, compliance to a specific method or manual) often present in clinical trials. Psychotherapy in clinical practice more often includes patients that would not be included in clinical trials depending on multiple psychiatric diagnosis, psychosocial problems or other problems that excludes them from clinical trials.

## Effects of Premature Dropouts

Premature dropout has been associated with a range of negative effects for both clients and therapists. In clinical trials, dropouts tend to report more dissatisfaction (Björk et al., 2009; Knox et al., 2011; Kokotovic & Tracey, 1987) and poorer treatment outcomes (Cahill et al., 2003; Klein et al., 2003; Lampropoulos, 2010; Pekarik, 1983, 1992; Swift et al., 2009), compared with therapy completers. Therapists are likely to experience loss of revenue (i.e., in private practice), and a sense of failure or demoralization when clients prematurely drop out (Barrett et al., 2008; Ogrodniczuk et al., 2005; Piselli et al., 2011).

## Factors Related to Premature Dropout

The therapeutic alliance has consistently been associated with outcome in psychotherapy (Horvath et al., 2011; Lambert & Barley, 2002; Safran et al., 2014). Research commonly shows that a strong alliance is related to better outcomes (Bickman et al., 2012; Flückiger et al., 2018; Spinhoven et al., 2007; Zuroff & Blatt, 2006), and that a weak alliance is related to dropout (Barrett et al., 2008; Sharf et al., 2010). Some meta-analyses show significant correlations between repairing ruptures in the therapeutic alliance and therapy outcome measured either as therapy completion, premature dropout or as change on symptoms measures (Eubanks et al., 2018; Safran et al., 2011; Safran et al., 2014). Moreover, the ability to manage behavioural, cognitive, somatic, and affective reactions during psychotherapy (related to the therapist’s own unresolved emotional stressful events or themes during therapy) may also influence psychotherapy process and outcome. The ability to manage and potentially use own reactions to “what happens during psychotherapy” – for example if the therapist has dealt with his/her own negative experiences and are aware of them - may increase the ability to effectively help the client (Hayes et al., 2011; Hayes et al., 2018).

Overall, the proportion of dropout reported in different studies is related to the definition of dropout, and since there is no consensus on the definition comparisons on rates is difficult. However, the literature suggests that dropout is common, has negative effects on clients and their therapists, and that a poor therapeutic alliance may increase the risk of a premature dropout.

The aim of this study was to investigate how common premature dropout is in clinical practice, to analyse perceived reasons behind a dropout, the role of therapeutic alliance and feelings associated with dropouts. Psychotherapists working with different orientations, target groups, and in different settings completed an online survey with the aim to reach a broad sample.

## Method

### Procedure

The study was conducted online using an anonymous questionnaire during 2 months in the spring of 2020. The study was announced via social media (Facebook), email to employees at two outpatient psychiatric clinics and networks for psychotherapists. In Sweden, where the study was conducted, almost all practicing clinicians have regular internet access. In total 594 persons accessed the website, and, of those, 116 persons (19.5%) completed the whole questionnaire.

### Data Analysis

The data from the survey were prepared with SPSS statistics version 26. Means (*M*), Medians (*Mdn*), Standard deviations (*SD*) and Ranges were calculated. ANOVAs were calculated to investigate differences between means. Nominal data were compiled and descriptive measures such as percentages were calculated.

### Participants

Participants were psychotherapists from different professional backgrounds. They had at least basic psychotherapy training (which in Sweden is 3 years) and used psychotherapeutic methods in their work. Participation was anonymous and no data was collected that could be used to identify the participant. Of the 116 psychotherapists who participated, 83 were female (70.9%). They had worked as psychotherapists for an average of 10.51 years (*SD* = 7.91). The professional background of the participants was: clinical psychologists (*n* = 67; 57.8%), social workers (*n* = 28; 24.1%), nurses (*n* = 4; 3.4%), medical doctors (*n* = 1; 0.9%) and other (*n* = 16; 13.8%). Regarding the therapists’ main methodological orientation, the following distribution was obtained (multiple answers were possible): cognitive behavioural therapy (CBT) (*n* = 99; 84.6%), Psychodynamic psychotherapy (PDT) (*n* = 47; 40.2%), Interpersonal psychotherapy (IPT) (*n* = 19; 16.2%), Family therapy (FT) (*n* = 11; 9.4%), Humanistic/Existential psychotherapy (*n* = 7; 6%) and 20 (*n* = 20; 17.1%). See Table 1 for further description of the participants.

**Table 1:** Background Data of the Participating Psychotherapists (N = 117)

Variables	*n* (%)
**Gender (female)**	83 (70.9%)
**Age in profession**	*M* = 10.51 years
Profession	
Psychologists	67 (57.8)
Social workers	28 (24.1)
Nurses	4 (3.4)
Medical Doctors	1 (0.9)
Others	16 (13.8)
Workplace	
Public sector (primary care)	46 (39.6)
Public sector (psychiatry)	43 (37.1)
Private sector (psychiatry)	11 (9.4)
Private sector (primary care)	13 (11.2)
Private practice	31 (26.7)
Other	6 (5.2)
Age group	
Children (0-13 years)	14 (12.1)
Youths (14-18 years)	21 (18.1)
Young adults (18-25 years)	35 (30.2)
Adults (18-65 years)	97 (83.6)
Older adults (65 years-)	14 (12.1)
Psychotherapeutic orientation	
Cognitive behaviour therapy (CBT)	99 (84.6)
Psychodynamic therapy (PDT)	47 (40.2)
Interpersonal psychotherapy (IPT)	19 (16.2)
Other	21 (17.9)

### Measures

A brief questionnaire was developed for use in the present study. The questionnaire was developed in discussion with clinicians, by consulting the literature on dropout and the therapeutic relationship in psychotherapy. To increase the content validity, the questionnaire was piloted with 6 colleagues, all licensed clinical psychologists, and researchers in clinical psychology. They filled out the questionnaire individually which was followed by a discussion which resulted in some adjustments and clarifications. The final questionnaire started with this definition:

The aim of this study is to explore psychotherapists’ clinically based opinion of the frequency of dropouts in psychotherapy and, also their feelings prior to and after a dropout. Our definition of dropout is “when a client stops coming to an agreed and started psychotherapy without notice.”

The initial part of the questionnaire consisted of 6 items on generic information regarding gender, years of working with psychotherapy, primary age group in the work, type of organization, professional background and use of psychotherapeutic methods. The scales were made as nominal variables where the most common professional backgrounds, organizations and most used psychotherapeutic methods were specified as single response options. There was also an open-ended alternative to capture alternatives that were not specified. The participants were asked to estimate the dropout rate in their therapies, based on our definition of a premature dropout, as a percentage of their total number of psychotherapies. In the next section participants were asked why they believed a typical dropout had occurred, their own feelings during the therapy and after the dropout. Further, feelings before and after the dropout were derived from a feeling checklist used in psychotherapy process research (Lindqvist et al., 2017). There were 20 different feelings which were rated on a five-point Likert-scale ranging from 1 (‘not very important for me’) to 5 (‘very important to me’). In the next part, the therapeutic alliance was rated with three items (task, goal, and emotional bond). These items were rated on a three-point Likert-scale from 1 (‘bad’) to 3 (‘very good’). Further the participants rated if they had suspected that the clients would drop out. The rating was made on a five-point Likert-scale ranging from 1 (‘not very important for me’) to 5 (‘very important to me’). Finally, questions regarding discussing the suspicion of a potential dropout with someone (yes/no/don’t know) and in that case with whom (e.g. supervisor, colleague, friend, partner), and lastly if they had received the support they needed in psychotherapy supervision. The time for filling out the form was approximately 15 minutes.

## Results

The average estimated dropout-rate, defined as the percentage of the total number of psychotherapies during the last two years was 8.89% (*Mdn* = 5, *SD* = 8.34, Range = 0–50%). We conducted an ANOVA-analysis to test if there were any differences regarding the estimated dropout rate between CBT, PDT, IPT and Eclectic therapy and no differences were found, all *p*-values were above *p* > .11. Ratings in the survey done with questions on Likert-scales generally generated responses in the middle of the scales, as measured by median. Few therapists rated in the top end of the scales (4 or 5). The views among the therapists were primarily that dropout depended on the clients, by for example not wanting to do specific interventions or not responding to certain interventions.

### Reasons for Dropout

Table 2 shows the therapist’s ratings of reasons for dropout (in the order of highest rating first).

**Table 2:** Therapists’ Ratings of Reasons for Dropout

Variables	*M*	*Mdn*	*SD*
The client did not want to do specific interventions related to the method.	3.08	3	1.10
The client did not “respond” to the intervention.	2.99	3	1.25
It seemed like the client did not believe that the method would help.	2.92	3	0.10
The client was in a difficult psychosocial situation.	2.79	3	1.21
The client had difficulties in the attachment with me (the therapist).	2.61	3	1.04
We had a weak emotional bond.	2.55	2	1.05
The client was discontent with me (the therapist).	2.47	2	0.90
The client had too complex psychological problems.	2.43	2	1.15
The therapy had low effect.	2.41	2	0.92
It was the wrong method for the problem.	2.38	2	0.92
We disagreed about the goals with the therapy.	2.31	2	0.97
I think we had too few sessions for our disposal.	2.28	2	1.32
I thought that the client was too difficult.	2.20	2	0.11
I (the therapist) had difficult to attach to the client.	2.08	2	0.92
It was the client’s age.	1.69	1	0.94
The client used drugs.	1.65	1	1.06
The client started another psychotherapy.	1.39	1	0.87

*Note. N* = 107. Instruction: *Think of a typical dropout, what do you think it was related to? (Mark one or several alternatives (1 not important and 5 very important).*

When using mean as measure the most common reason for a dropout was that the client did not want to perform the intervention, respond to it, or did not believe in it. The lowest ratings were reasons for dropout related to clients age, clients using drugs or had started another therapy.

### Emotions Related to Dropout

The therapists were asked to rate their feelings *during* the therapy and *after* the dropout. Table 3 shows the rating of feelings *during* therapy as indicated by the therapists. 

**Table 3:** Rating of Feelings During Therapy as Indicated by the Therapists

Variables	*M*	*Mdn*	*SD*
Interested	2.94	3	0.67
Calm	2.52	3	0.72
Energetic	2.31	2	0.76
Insecure	2.28	2	0.81
Sceptical	2.13	2	0.84
Powerless	2.07	2	0.82
Content	2.01	2	0.68
Irritated	2.01	2	0.78
Worried	1.97	2	0.77
Tired	1.96	2	0.85
Neutral	1.94	1	0.86
Disappointed	1.93	2	0.73
Tense	1.90	2	0.84
Surprised	1.84	2	0.79
Shame	1.70	2	0.78
Overwhelmed	1.62	1	0.81
Relieved	1.58	1	0.67
Bored	1.54	1	0.72
Angry	1.54	2	0.69

*Note.* *N* = 107. Instruction: *If you think of the same therapy, which of the following emotions did you experience* ***during*** *therapy, as you remember it? For example “I felt…..” (1 not important and 4 very important). Mark one or several feelings.*

Feelings with the highest mean reported by the therapists during therapy were interested, calm, and energetic. Feelings with the lowest mean were relieved, bored, and angry.

In Table 4, the therapists’ feelings following a dropout are presented.

**Table 4:** Therapists’ Feelings Following a Dropout

Variables	*M*	*Mdn*	*SD*
Self-doubt	2.79	3	1.04
Touched	2.66	3	1.01
Powerless	2.61	2	1.13
Disappointed	2.52	2	0.96
Calm	2.38	2	1.12
Surprised	2.22	2	1.08
Annoyance at the client	2.13	2	0.87
Doubt regarding my method	2.12	2	1.06
Annoyance at myself	2.10	2	1.03
Guilt	2.10	2	1.06
Relieved	1.96	2	0.94
Worried	1.93	2	1.00
Shame	1.92	2	0.96
Neutral	1.73	1	0.98
Indifference	1.39	1	0.72
Satisfied	1.39	1	0.66
Overwhelmed	1.37	1	0.78
Bored	1.28	1	0.69
			

*Note.* *N* = 107. Instruction: *What did you feel after the dropout? I felt…. Mark one or several options. (1 not important and 5 very important).*

The feelings with highest mean after dropout was self-doubt, being touched and powerless. Feelings with lowest mean were satisfied, overwhelmed, and bored.

### Therapeutic Alliance and Dropout

The therapeutic alliance with the client who dropped out in mind was rated by the therapists using an ordinal scale with three response options (bad, good, very good). Ratings of alliance in association with a dropout therapy are presented in Table 5.

**Table 5:** Ratings of Alliance in Association With a Dropout Therapy

Items	Low	Good	Very good	*M*	*Mdn*	*SD*
The task of the therapy	32%	55%	13%	1.81	2	0.65
The goal in the therapy	26%	64%	10%	1.84	2	0.58
Emotional bond in the therapy	33%	55%	12%	1.79	2	0.64

*Note.* *N* = 107. Instruction: *Afterwards, how would you rate the therapeutic alliance between you and the client who dropped out? (Rate between 1-3 were 1 is low and 3 is very good).*

All three dimensions (task, goal, and bond) of the alliance were generally rated as good, with few (10-13%) stating that it was very good. One third rated the alliance in all three dimensions as low. There was no significant difference between the different aspects of the therapeutic alliance, *F*(2, 105) = .24, n.s.).

### Support From Others Regarding Suspicions About Dropout

Regarding the question if the therapists had suspected the dropout during therapy, 24% did not suspect dropout while 76% had suspected dropout. About one fourth (23%) of the therapists had talked with their clients about their suspicions, 37% of the therapists did not talk to the client and 40% did not remember. About 59% of the therapists had talked to a supervisor or a colleague when they suspected that their client would drop out. Only one third (30%) felt that they had received support.

## Discussion

One aim of this study was to explore the extent of premature dropout in clinical practice as rated by therapists. The estimated dropout for the last two years was 8.89%. The results indicate that in clinical practice the dropout-rate, as defined in this study, is lower than in earlier studies in which the estimated dropout-rate has been 20% or higher (Swift & Greenberg, 2014). As mentioned in the introduction, Wierzbicki and Pekarik (1993) estimated the dropout-rate to be 47% based on 125 studies, whereas Swift and Greenberg (2012) reported a dropout rate of 19.7% in their meta-analytic study of 669 research studies. It is important to note that these discrepancies most likely depend on the difference in definition of dropout used in studies and reviews. Regarding studies on differences between psychotherapy orientations a significant difference has been reported in depression studies in which CBT was found to result in more dropouts than other therapies (Cuijpers et al., 2008). Swift and Greenberg (2014) reported that that there may be differences between psychotherapies related to diagnosis and that depression, eating disorders and PTSD may be associated with differential dropout rates. These differences were *not* investigated in our study, but we cannot exclude that the sample we obtained and the groups of clients and/or psychotherapy method the therapists worked with influenced the estimated dropout rate.

Information about the proportion of dropout in regular clinical practice seems to be scarce. Cinkaya (2016), in a study on outpatients in Germany, reported that patients with personality disorder were most likely to drop out whereas patients with depression, somatoform, and anxiety disorder were less likely to drop out. Although the estimation done by the therapists in this study could be biased and uncertain, our findings is relevant for the understanding of how common dropout is in clinical practice.

Overall, some prior studies have reported substantially higher dropout rates than we found in this study. There are some possible explanations. First, we used a definition that leaves out agreed upon terminations that would have been regarded as dropouts in research studies. Another possibility, again referring to the difference between research studies and clinical settings, it that the length of a therapy and the demands on the client may be more flexible in clinical settings than in research studies in which for example the number of therapy sessions tend to be tied to treatment manuals. However, this does not mean that the figure we found is low. If almost one out of ten client dropout without any discussion or agreement it is still a problem in clinical settings both for the client and the service provider.

Our study explored reasons and feelings related to a typical premature dropout and the perception of the therapeutic alliance in such therapies. Based on means, the three most common emotions *during* therapy were *interested*, *calm*, and *insecure*. *After* the dropout the three most common emotion were *self-doubt*, *touched* and *powerlessness*. Our results indicate that premature dropouts affect the therapists negatively. After premature dropout therapists tend to feel self-doubt and experience emotions like *powerlessness*. On the other hand, the most common reasons for dropout stated by the therapists were that the client did not want to perform the intervention, respond to it, or did not believe in it. It appears as if the therapists blame themselves emotionally but rationally blame the client. Another explanation might be that therapists do not manage to convince their client of about the ways in which they are supposed to work in therapy and therefore feel powerless in relation to what they are supposed to do in therapy, agreement about goals in therapy, or own conviction about what is best for the client.

Overall, the therapists rated the therapeutic alliance as good. Approximately 30% rated the alliance as low regarding agreement on tasks and the emotional bond, and 26% rated the alliance as regarding goal. The result is a bit puzzling because it would be expected that maybe a higher percentage would rate the alliance as low or weak. As mentioned in the introduction, research has consistently showed that a strong alliance is related to good outcomes (Bickman et al., 2012; Spinhoven et al., 2007; Zuroff & Blatt, 2006), and that a weak alliance is related to dropout (Barrett et al., 2008; Sharf et al., 2010). Some meta-analysis showed a significant correlation between repairing the alliance and therapy outcome (Safran et al., 2011; Safran et al., 2014), which we did not study but could be important to investigate in relation to dropout in future research. Another possibility would be to investigate ruptures in the alliance, which also have been associated with treatment outcome (Larsson et al., 2018). In a micro-analysis of sessions before a dropout more withdrawal alliance ruptures were observed (Gülüm et al., 2018). Findings also suggested that both therapists and clients decreased the pace of work and engaged in less exploration during the sessions before the dropout (Gülüm et al., 2018). Our findings correspond with these findings as approximately 30% rated the alliance as low and suspected a dropout. The fact that they mostly did not talk to the client suggests a withdrawal pattern in the therapeutic alliance. Nissen-Lie et al. (2017) found that when therapists actively help clients deal with clinical problems by exercising reflexive control and problem solving, it was associated with positive change while avoiding problems was associated with less change. It seems reasonable to assume that when clients dropout, they do so because they experience that they are not getting the help they hoped for or do not have enough trust in the therapist being able to be helping them sufficiently. The therapists provided the highest ratings for the following reasons: a) the client did not want to engage in or respond to specific interventions, b) the clients did not seem to believe that the method would help them. The discrepancy between what therapists reported as reasons for the dropout and their own feelings during therapy suggests that the client and the therapist have different experiences related to therapy. One example of this would be that the therapist is interested and eager to help but the client do not want to engage in or even resists interventions. It is likely that relational strains, which may be interpreted as a rupture in the therapeutic alliance, affects therapy negatively. If the rupture is not articulated there may be a silent withdrawal rupture in the therapeutic alliance, It may also be that psychological mechanisms (for example countertransference or avoidant coping) may be involved without the therapist necessarily being aware of it and still communicating these sentiments in the therapy. Another possibility in terms of psychological mechanisms is when we suspect that a client will leave therapy and this suspicion triggers anxiety about being inferior, being left in other relationships, not being “good-enough”, a failure or other signs of downgrading our competence or even ourselves as persons. Thoughts and emotions like this are hard to verbalize and therapists may hesitate to reveal to the client that that he/she suspect that the client will leave the therapy. Our findings showed that a majority suspected premature drop out but only 23% of the therapists had communicated about their suspicions with the client. It seems like many therapists suspect a dropout, but do not communicate their suspicions.

### Limitations and Strengths

The study has several limitations. First, the recruitment of therapists was done on the internet via Facebook, email to psychiatric outpatient clinics and different psychotherapy networks. This narrowed down the sample to persons frequently using the internet (e.g. social media and online networks) and could be reached. Even if the sample was limited by the number of persons who could answer the questionnaire, we still believe we reached a fairly broad sample and that many currently active psychotherapists use the internet and social media. Using a postal survey or telephone interview could possibly lead to different estimates and findings even if we doubt there would be major discrepancies. Further, although we asked the therapists to think of a particular premature dropout it is difficult to know if the answers reflect a single dropout or if they rather mirror general opinions related to non-agreed premature dropouts. It can be hard to remember specific emotions or what was going on in hindsight, and we cannot exclude memory bias and selective reporting. Cuijpers et al. (2015) also showed that there were differences in how dropout had been defined which makes it difficult to interpret the findings.

Some strengths with our study are that we measured what therapists clinically encounter in association with dropouts. Further, the observation that clients drop out fairly often most likely reflects what occurs in a typical clinical setting and adds information to what is already known regarding research and educational settings for psychotherapy where most studies regarding estimated dropout rates have been conducted. Finally, the respondents were from different organizations, used therapeutic methods and had varied work experience as therapists.

### Future Research

This study indicates that there are discrepancies in the number of premature dropouts observed in clinical settings, research studies, and studies made in psychotherapy education settings. However, the number of people in the general population who have an experience of premature dropout from psychotherapy is to our knowledge not known and could be investigated as was done long ago with regards to therapy experiences in the Consumers Report study (Seligman, 1995). It is reasonable to assume that there are different reasons behind premature dropouts. To investigate reasons for premature dropout it will be vital to ask clients about their reasons for terminating therapy. To further investigate therapists’ views on the impact of dropout, interviews or focus groups are possible methods to obtain a deeper understanding of processes related to dropout. By analysing video clips of therapy session in which clients subsequently dropout, one could gain a deeper understanding the reasons for and the process of dropout.
